# Sequence Read Depth Analysis of a Monophyletic Cluster of Y Chromosomes Characterized by Structural Rearrangements in the AZFc Region Resulting in DYS448 Deletion and DYF387S1 Duplication

**DOI:** 10.3389/fgene.2021.669405

**Published:** 2021-04-16

**Authors:** Francesco Ravasini, Eugenia D’Atanasio, Maria Bonito, Biancamaria Bonucci, Chiara Della Rocca, Andrea Berti, Beniamino Trombetta, Fulvio Cruciani

**Affiliations:** ^1^Laboratory Affiliated to Istituto Pasteur Italia-Fondazione Cenci Bolognetti, Dipartimento di Biologia e Biotecnologie “Charles Darwin”, Sapienza Università di Roma, Rome, Italy; ^2^Istituto di Biologia e Patologia Molecolari, Consiglio Nazionale delle Ricerche, Rome, Italy; ^3^Sezione di Biologia, Reparto CC Investigazioni Scientifiche di Roma, Rome, Italy

**Keywords:** Y chromosome, Y-STR, AZFc, genomic rearrangement, infertility, forensic genetics

## Abstract

The azoospermia factor c region (AZFc), located in the long arm of the human Y chromosome, is frequently involved in chromosome rearrangements, mainly due to non-allelic homologous recombination events that occur between the nearly identical sequences (amplicon) that comprises it. These rearrangements may have major phenotypic effects like spermatogenic failure or other pathologies linked to male infertility. Moreover, they may also be relevant in forensic genetics, since some of the Y chromosome short tandem repeats (Y-STRs) commonly used in forensic analysis are located in amplicons or in inter-amplicon sequences of the AZFc. In a previous study, we identified four phylogenetically related samples with a null allele at DYS448 and a tetrallelic pattern at DYF387S1, two Y-STRs located in the AZFc. Through NGS read depth analysis, we found that the unusual Y-STR pattern may be due to a 1.6 Mb deletion arising concurrently or after a 3.5 Mb duplication event. The observed large genomic rearrangement results in copy number reduction for the RBMY gene family as well as duplication of other AZFc genes. Based on the diversity of 16 additional Y-STRs, we estimated that the duplication/deletion event occurred at least twenty generations ago, suggesting that it has not been affected by negative selection.

## Introduction

**FIGURE 1:**
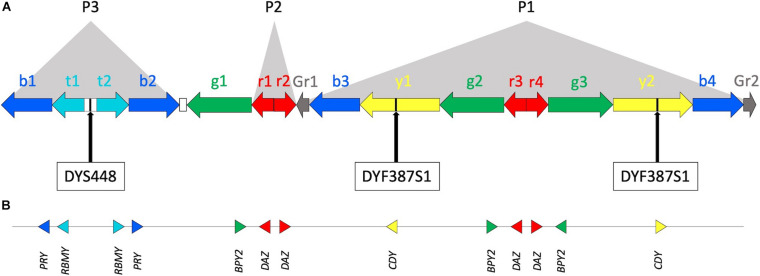
The Y chromosome AZFc region. **(A)** Multiple copies of six amplicons are represented by color-coded arrows (b—blue, t—teal, g—green, r—red, y—yellow, and Gr—gray; adapted from Kuroda-Kawaguchi et al., 2001; Teitz et al., 2018, not in scale). Arrows direction indicates amplicon copy orientation. Most of the amplicons are arranged to form P1, P2, and P3 palindromes. The white rectangles between t1, t2, and b2, g1 represent the large P3 spacer and a single copy of Inverted Repeats 1 (IR1), respectively. Approximate positions of DYS448 and DYF387S1 short tandem repeats are shown. **(B)** Locations of protein coding genes within the AZFc. Triangle orientation refers to 5’–3’ polarity.

Y chromosome short tandem repeats (Y-STRs) are commonly used in forensic genetics (Kayser, 2017). They are especially useful for kinship analysis of the male-lineage and for personal identification from female-male DNA mixtures, such as those collected from sexual assaults (Gusmão et al., 2006; Andersen and Balding, 2019). Some of the Y-STRs included in forensic multiplexes are located in the azoospermia factor c (AZFc) region (Figure 1). This region, covering a total of 4.4 Mb, is made up mostly by duplicated sequences (known as amplicons), which exhibit a high sequence identity and contain male-specific genes necessary for spermatogenesis (Figure 1; Teitz et al., 2018). Most of the Y amplicons are arranged in large palindromic structure that can contain one or more pairs of amplicons (Kuroda-Kawaguchi et al., 2001). Due to the high sequence identity, non-allelic homologous recombination (NAHR) events occur frequently between the amplicons and can lead to chromosome rearrangements (Repping et al., 2003; Teitz et al., 2018). Several rearrangements of this region have been reported and they can have remarkable phenotypic effects: the complete deletion of AZFc is linked to spermatogenic failure (Vogt et al., 1996; Kuroda-Kawaguchi et al., 2001; Bansal et al., 2016; Krausz and Casamonti, 2017; Zhou et al., 2019), while less extended deletions or duplications can be correlated with male reduced fertility and other pathologies (Lynch et al., 2005; Nathanson et al., 2005; Giachini et al., 2009; Lange et al., 2009).

Recently, it has been demonstrated that two or more recombinational events may occur within the AZFc in the same Y chromosome lineage and that the resulting pattern may be maintained by natural selection (Teitz et al., 2018). Indeed, after a deletion event, a duplication one can in part restore the proper gene dosage for the male-specific multi-copy genes.

When using Y-STRs, AZFc rearrangements can be relevant also for forensic purposes. They are of critical significance when there is the need to discriminate between one or more contributors, like in rape cases. Indeed, in Y-STR profiles, finding extra peaks in a locus may represent two different situations: either a Y-chromosomal rearrangement involving this locus or the presence of multiple (related) contributors in the sample. Furthermore, duplications or deletions can be useful to distinguish between different individuals with similar haplotypes.

Two Y-STRs comprised in the widely used forensic Yfiler^TM^ Plus PCR amplification kit (ThermoFisher Scientific) can be found in the AZFc: DYS448, located in a unique sequence between amplicons teal1 (t1) and teal2 (t2) (i.e., P3 palindrome spacer), and the multi-locus DYF387S1, present in two copies in amplicons yellow1 (y1) and yellow2 (y2) (Figure 1).

Several instances of duplications or deletions in these STRs have been previously reported. The DYS448 duplication is common in Portuguese individuals of African descent (Dente et al., 2019), it has been found in the Italian population (Turrina et al., 2015) and it has also been observed in several African subjects of haplogroup E1a-M33 (Balaresque et al., 2008; D’Atanasio et al., 2019; Della Rocca et al., 2020). Null alleles of DYS448 caused by complete deletion of the locus (or, in some cases, by point mutations at primer binding sites) have been reported in different populations (Balaresque et al., 2008; Budowle et al., 2008; Turrina et al., 2015; Westen et al., 2015; Shonhai et al., 2020). The diallelic locus DYF387S1 is frequently involved in duplication events, resulting in triallelic or tetrallelic patterns (Willuweit and Roewer, 2015). Recently, several Copy Number Variants (CNVs) of DYF387S1 caused by duplications, deletions and gene conversion were found within the Japanese population with different distribution among Y chromosome haplogroups (Watahiki et al., 2020).

In a previous study, we identified, through Yfiler^TM^ Plus genotyping, four phylogenetically related African individuals of the same Y chromosome haplogroup (R1b–V515) that shared a null allele in DYS448 (Della Rocca et al., 2020). Three of these subjects (M4–85, M4–98 and M4–100) also showed a DYF387S1 triallelic pattern with the same alleles. Nevertheless, the Relative Fluorescence Units (RFU) values for these individuals suggested that the apparent triallelic pattern was indeed a hidden tetrallelic pattern, i.e., a duplication of both copies of DYF387S1 followed by a one-repeat mutation in one of the copies (Della Rocca et al., 2020). The fourth subject (M4–106), although showing a diallelic DYF387S1 pattern, was characterized by atypical RFU values compared to other Y-STR loci, a situation compatible with a full DYF387S1 duplication with identical alleles in pairs. Therefore, the events which caused this condition are likely to have occurred in an ancestral Y chromosome from which the four subjects originated (Della Rocca et al., 2020).

In this study we report the molecular characterization of the AZFc region of one of these subjects (M4–98) through comparative NGS read depth analysis. Thus, we reconstructed the possible pattern of duplication and deletion that involved these Y-STR markers.

## Materials and Methods

### DNA Samples

The subject analyzed (M4–98) was sampled in Cameroon. Of the two control subjects, one was collected in Cameroon (W005) and the other in Europe (W043) (Supplementary Table 1). The other three subjects that show the particular Y-STRs pattern (M4–85, M4–100, M4–106) and the subjects included for network construction and time estimates (belonging to haplogroup R1b–V69 which comprises haplogroup R1b–V515) were collected in Cameroon, Chad, Libya and Egypt (Supplementary Tables 1, 2). For all the samples in this study (except for W005 and W043), Y-STRs and Y-SNP analyses were previously performed (D’Atanasio et al., 2019; Della Rocca et al., 2020). For each subject, the ethnic identity was assessed by self-identification. Blood or saliva (using the non-invasive Oragene collection kits) was collected from participants, after appropriate informed consent, and DNA was extracted using standard protocols and following manufacturer’s instructions. The research project was formally approved by the “Sapienza Università di Roma” Ethical Committee (Document number 2755/15).

### Y-STR Multiplex Genotyping

Y-STR genotyping of all the R1b–V69 samples was conducted in previous studies (D’Atanasio et al., 2019; Della Rocca et al., 2020) using the Yfiler^®^ Plus amplification kit (ThermoFisher Scientific), according to the manufacturer’s protocol.

### Whole Genome Sequencing

Whole genome sequencing was performed for one of the samples with the particular Y-STRs pattern (M4–98) and for two control samples (W005 and W043). We sequenced only one sample showing DYS448 null allele and DYF387S1 duplication since we assumed that the events that lead to this pattern have occurred in an ancestral Y chromosome from which the four phylogenetically related subjects originated (M4–85, M4–98, M4–100, M4–106). We sequenced two control in order to be sure that we can observe and make a comparison with the normal situation of the AZFc, since chromosomal rearrangements in this region are frequent.

Library preparation, sequencing and alignment were performed by BGI-Tech (Hong Kong). After random fragmentation and amplification by ligation-mediated PCR, the rolling circle amplification was performed to produce DNA Nanoballs (DNBs). Pair-end reads were sequenced on the DNBseq platform subsequently to the loading of the qualified DNBs into the patterned nanoarrays. Base-calling was performed with DNBseq basecalling Software with default parameters. The sequences obtained were aligned to the human reference genome (GRCh37/hg19) with Burrows-Wheeler Aligner (BWA) software (Li and Durbin, 2009). For the samples analyzed through NGS (Supplementary Table 1), whole genome sequencing was performed at an average sequencing depth of 38.7×, 36.4× and 41.8× for M4–98, W043 and W005, respectively.

### Amplicon Copy Number Variation (CNV) Detection

To detect variation in the number of copies of each amplicon we used the method developed by Teitz et al. (2018), normalizing the mean depth of an amplicon by the mean depth of a 1-Mb single copy region of the same Y chromosome (see Supplementary Table 3 for positions and lengths of the sequences analyzed). In this way, an amplicon with the reference copy number shows a normalized depth of 1, while an amplicon CNV has a different value depending on the number of copies. An amplicon CNV has been called if the normalized depth value exceeds the threshold set by the midpoints between the expected depth values of each amplicon. For instance, in a male with two copies of the teal amplicon, like the reference, the expected normalized depth value is 1 (2/2), while in a male with one copy is 0.5 (1/2) and in a male with zero copies is 0 (0/2). Thus, a male will be called as having one copy if the normalized depth value is between 0.25 and 0.75, while he will be called as having zero copies if the value is below 0.25 (Teitz et al., 2018). To check whether the null allele in DYS448 is due to a complete deletion, we performed the same analysis also for P3 spacer.

To confirm the results obtained with this method, we developed an alternative method based on the Exponential Moving Average (EMA). We calculated the EMA in the AZFc region with 50 kb sliding windows 1-bp moving using the R package “TTR” for each sample. We then normalized the EMA values of M4–98 by the EMA values of the control samples. Although values may be fluctuating due to casual differences in sequencing, a normalized value of ≈1 within an amplicon indicates the reference copy number, while other values denote copy number variations. We visualized this analysis with a linear graph made with the R package “ggplot2.”

### Network Construction and Time Estimates

We used the Network 5.0 software to reconstruct a median joining network representing the phylogenetic relations among the Yfiler^®^ Plus haplotypes of the Y chromosome haplogroup R1b-V69, which comprises the subjects analyzed of haplogroup R1b-V515. For this analysis, we removed four complex loci: the two multi-copy loci DYS385 and DYF387S1, DYS448 which shows null alleles in analyzed samples and the interrupted marker DYS389II.

Time estimates were obtained using the ASD (Average Square Distance) method from the markers included in the Yfiler^®^ Plus kit, after removing the rapidly mutating Y-STRs and the complex loci as in D’Atanasio et al. (2019); in addition, we also removed DYS448 because of its deletion, for a total of 16 loci used for the ASD calculation. The mutation rate of 3.18 × 10^–3^ per marker per generation used for time estimates was obtained averaging the known germline mutation rates for the 16 loci (Ballantyne et al., 2010, 2012).

## Results

### Identification of NAHR Events

Since recombinational events are frequent in the AZFc region, we hypothesized that the DYS448 null allele and the apparent DYF387S1 triallelic pattern were due to chromosome rearrangements caused by NAHR events. To test this hypothesis, we used a sequencing-based approach to uncover the putative recombinational events.

Normalized depth values calculated with the method developed by Teitz et al. (2018) and the corresponding ratio between sample copy number and reference copy number for each amplicon and for P3 spacer are shown in Table 1. In subject M4–98, yellow (y) amplicon and P3 spacer are clearly duplicated and deleted, respectively. Indeed, y amplicon normalized depth value is ∼2, which means that the sample has twice as many copies as the reference, while P3 spacer normalized depth value is ∼0, meaning that this sequence is not present in the sample. This result is in agreement with M4–98 Y-STR profile: deletion of DYS448 locus (located in P3 spacer) and duplication of the two DYF387S1 loci (located in y amplicon). Other amplicons show different copy number variations. Blue (b) and gray (Gr) amplicons exhibit reference copy number (normalized depth value ∼1), while both copies of teal (t) amplicon are deleted (normalized depth value ∼0) and green (g) and red (r) amplicons have two more copies than the reference (normalized depth value ∼1.66 and 1.5, respectively). We obtained similar results with the normalized EMA analysis (Supplementary Figure 1).

**TABLE 1:** Normalized depth values calculated with the method of Teitz et al. (2018) and the corresponding amplicon sample copy number/reference copy number ratio for each amplicon and P3 spacer in M4–98, W043, and W005.

Subject	Amplicon	Normalized depth	Sample copy number/reference copy number ratio
M4–98	b	0.983	4/4
	t	0.076	0/2
	g	1.589	5/3
	r	1.475	6/4
	y	1.962	4/2
	Gr	0.966	2/2
	P3 spacer	0.022	0/1
W043	b	1.023	4/4
	t	1.021	2/2
	g	1.004	3/3
	r	0.965	4/4
	y	1.001	2/2
	Gr	1.024	2/2
	P3 spacer	1.010	1/1
W005	b	0.964	4/4
	t	0.966	2/2
	g	0.940	3/3
	r	0.969	4/4
	y	0.979	2/2
	Gr	0.962	2/2
	P3 spacer	0.959	1/1

**FIGURE 2:**
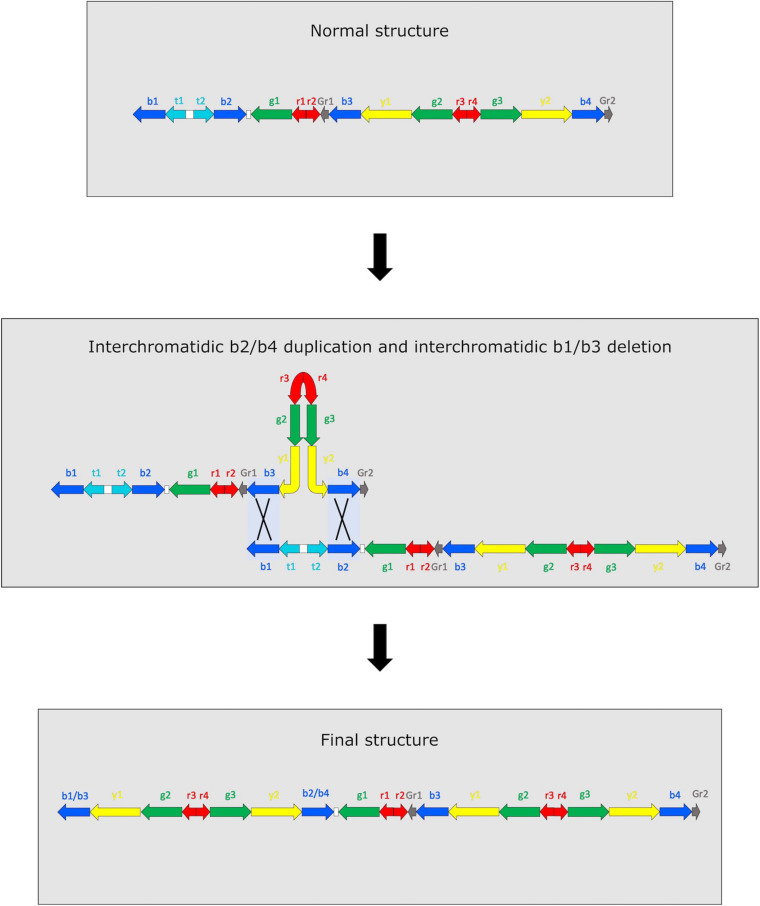
Representation of the two simultaneous NAHR events that may have led to DYS448 deletion and DYF387S1 duplication. Since it is not possible to determine which part of the amplicon copy remains after the recombinational events, they are indicated as hybrid copies (b2/b4 and b1/b3). The not observed recombinant chromatid is not represented.

Amplicons copy number in M4–98 sample is compatible with two different and mutually exclusive scenarios (Figure 2 and Supplementary Figure 2). The first scenario involves two concurrent NAHR events occurring within AZFc amplicons: an interchromatidic b1/b3 deletion and an interchromatidic b2/b4 duplication (Figure 2). The other possible scenario includes two independent and subsequent events (Supplementary Figure 2). The first event is an interchromatidic b2/b4 duplication, which has been frequently reported in different Y chromosome lineages (Repping et al., 2003; Teitz et al., 2018; Liu et al., 2021), while the second event is an intrachromatidic or interchromatidic b1/b3 deletion, which has also been previously reported in several Y haplogroups (Repping et al., 2003; Kuroda et al., 2018; Liu et al., 2020, 2021). A different succession of events is not able to explain the observed copy number variation. For example, if the deletion was the first event occurred, no b2/b4 duplication event would be possible in the resulting chromosome. Although it is not feasible to determine the precise positions in which these NAHR events occurred using sequencing data, it is possible to determine the approximate length of the sequences involved considering the positions of the amplicons, i.e., 3.5 Mb for the duplication and 1.6 Mb for the deletion. Notwithstanding in a previous study it has been possible to directly observe intermediate situations when two or more NAHR events occur in AZFc based on the Y chromosome phylogeny (Teitz et al., 2018), in our case we found no compatible intermediate: none of R1b-V69 samples shows only a DYF387S1 triallelic or tetrallelic pattern, i.e., the mark for a b2/b4 duplication.

### Network and Time Estimate Results

**FIGURE 3:**
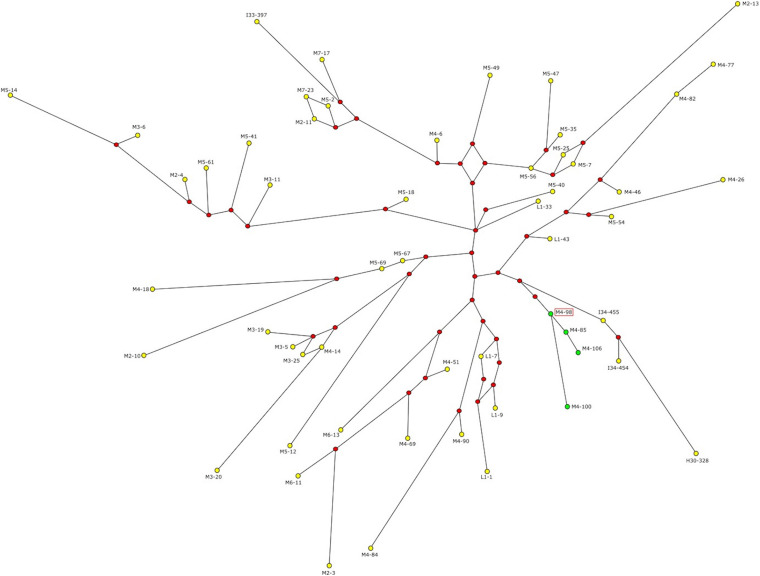
Network representing the relationship between R1b and V69 subjects. Green dots represent the four subjects carrying the genomic rearrangement, yellow dots all the remaining R1b-V69 subjects (dot area is proportional to the haplotype frequency). Red dots represent the median nodes of the network. The sequenced sample is highlighted by a red frame. Time estimates in years obtained with the ASD method.

Since the four subjects carrying the AZFc rearrangement belong to the same Y haplogroup and the same ethnic group, NAHR events are likely to have occurred only once in the recent past. In order to test this hypothesis and to estimate the time of the events, we defined the phylogenetic relationships among these subjects and the other R1b-V69 individuals through network construction with Y-STR loci (Figure 3). Although DYS448 and DYF387S1 were removed from the analysis due to their deletion or complexity, the four subjects (M4–85, M4–98, M4–100 and M4–106) form a homogeneous and apparently monophyletic cluster. Therefore, NAHR events probably occurred once in the branch that groups this cluster. To estimate when the events took place, we calculated with the ASD method the formation time of this cluster and the coalescence time of the upstream node, comprising our samples under examination and the samples phylogenetically closest to them (I34–454, I34–455, and H30–328) (Figure 3). We obtained a coalescence time of 492 ± 74 years ago for our cluster and a coalescence time of 2,738 ± 410 years ago for the upstream node. Thus, NAHR events must have occurred in between these two dates.

## Discussion

In this study we reported the molecular characterization through NGS read depth analysis of two recombinational events occurring within the AZFc region. NGS-based methods are increasingly used to detect CNVs in Y chromosome AZF regions and can replace the conventional STS-PCR method for both research and clinical purposes (Teitz et al., 2018; Liu et al., 2021).

Previously it has been demonstrated that amplicon copy number variation in AZFc is constrained by natural selection, because most of CNVs within this region are counter-selected (Teitz et al., 2018). A deletion event is likely to have worse effects than a duplication one on male reproductive fitness, since losing male specific genes relevant for spermatogenesis may potentially impair this process. Therefore, it is not surprising that it has been frequently observed, through Y chromosome phylogeny, duplications arising after deletion events that can restore most or all amplicon copy numbers and thus the genes within them (Teitz et al., 2018). However, the opposite sequence of events is also possible since gene duplication may alter the correct gene dosage resulting in reduced male fertility. A deletion that completely rescues a duplication can be difficult if not impossible to observe, because it can leave no sign on the rescued Y chromosome. Nevertheless, it is possible that a complete duplication rescue is not necessary to restore the correct gene balance for spermatogenesis. We argue that the pattern we have observed, i.e., a b1/b3 deletion arisen subsequently a b2/b4 duplication, can be a successful way to overcome an imbalance in male specific gene dosage or at least it is a status that does not significantly reduce the fitness of the individuals carrying it. Indeed, of the five gene families involved in these recombinational events (PRY, RBMY, BPY, DAZ, and CDY present in b, t, g, r, and y amplicon, respectively) the only that faced a reduction in copy number (from six to four copies) is RBMY, while other gene families either acquired two additional copies (BPY, DAZ, and CDY) or remained unaltered (PRY). Although the reduction in the number of RBMY genes has been linked to reduced sperm motility, men with four copies of RBMY can still be fertile (Yan et al., 2017). Accordingly, we observed that this rearrangement is conserved in a Y chromosome lineage that has persisted unaltered for at least 500 years. To the best of our knowledge, this is the first time that such a complex AZFc structural variation has been reported.

## Data Availability Statement

The data presented in this study are deposited in the European Nucleotide Archive (ENA, https://www.ebi.ac.uk/ena), accession number PRJEB43712.

## Ethics Statement

The studies involving human participants were reviewed and approved by “Sapienza Università di Roma” Ethical Committee (Document Number 2755/15). The patients/participants provided their written informed consent to participate in this study.

## Author Contributions

FC and BT conceived the study. FR, ED’A, MB, and BB performed the bioinformatic analysis. CD performed Y-STR genotyping. FR, ED’A, AB, BT, and FC discussed the results. FR, ED’A, and FC wrote the manuscript. All authors contributed to the article and approved the submitted version.

## Conflict of Interest

The authors declare that the research was conducted in the absence of any commercial or financial relationships that could be construed as a potential conflict of interest.
