# Supplementary figures and images for: Sequence Read Depth Analysis of a Monophyletic Cluster of Y Chromosomes Characterized by Structural Rearrangements in the AZFc Region Resulting in DYS448 Deletion and DYF387S1 Duplication
Source: Front Genet. 2021 Apr 16;12:669405. doi: 10.3389/fgene.2021.669405 (PMC8085532; doi:10.3389/fgene.2021.669405)

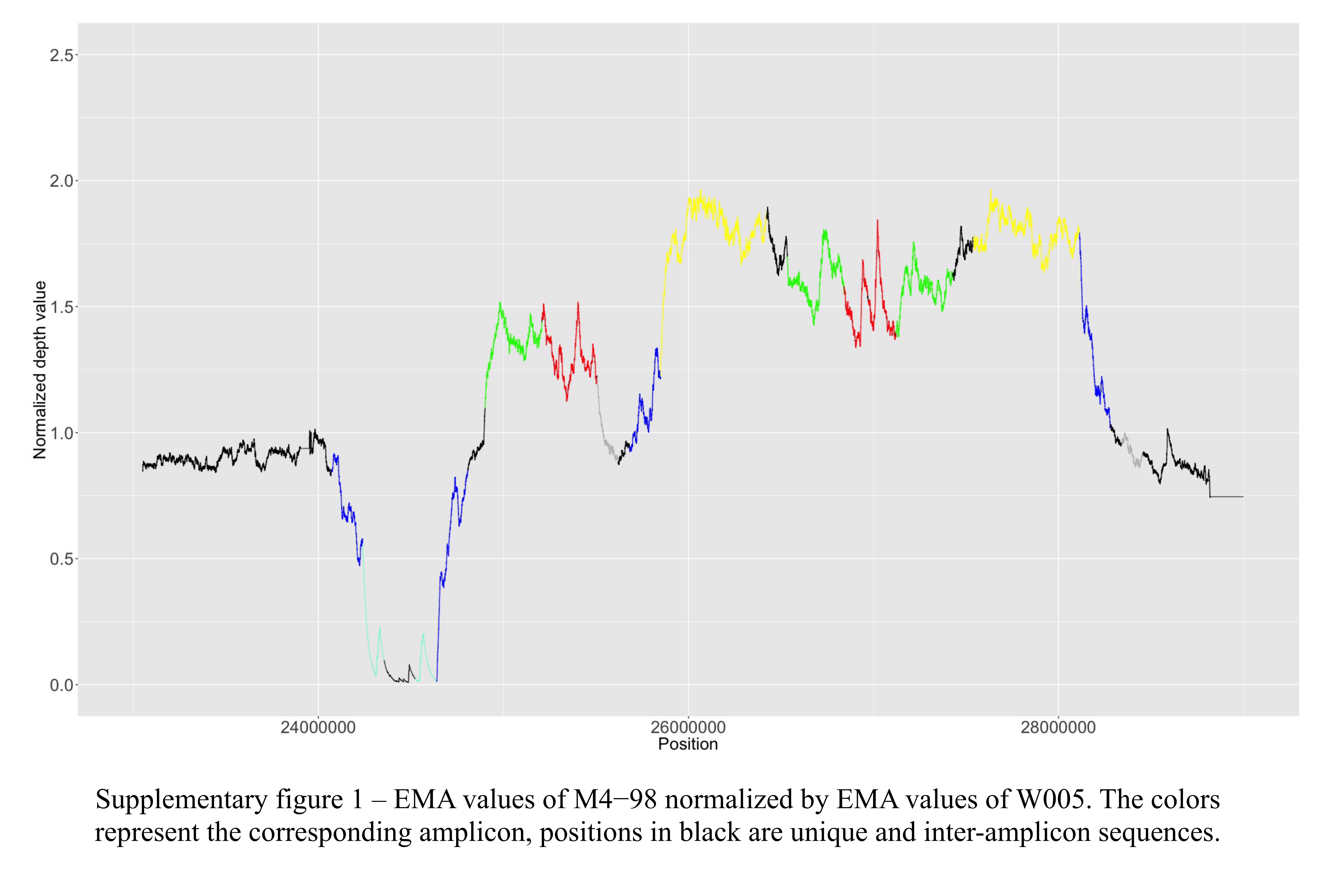

Supplement: Supplementary file 2 [file Image_1.JPEG]

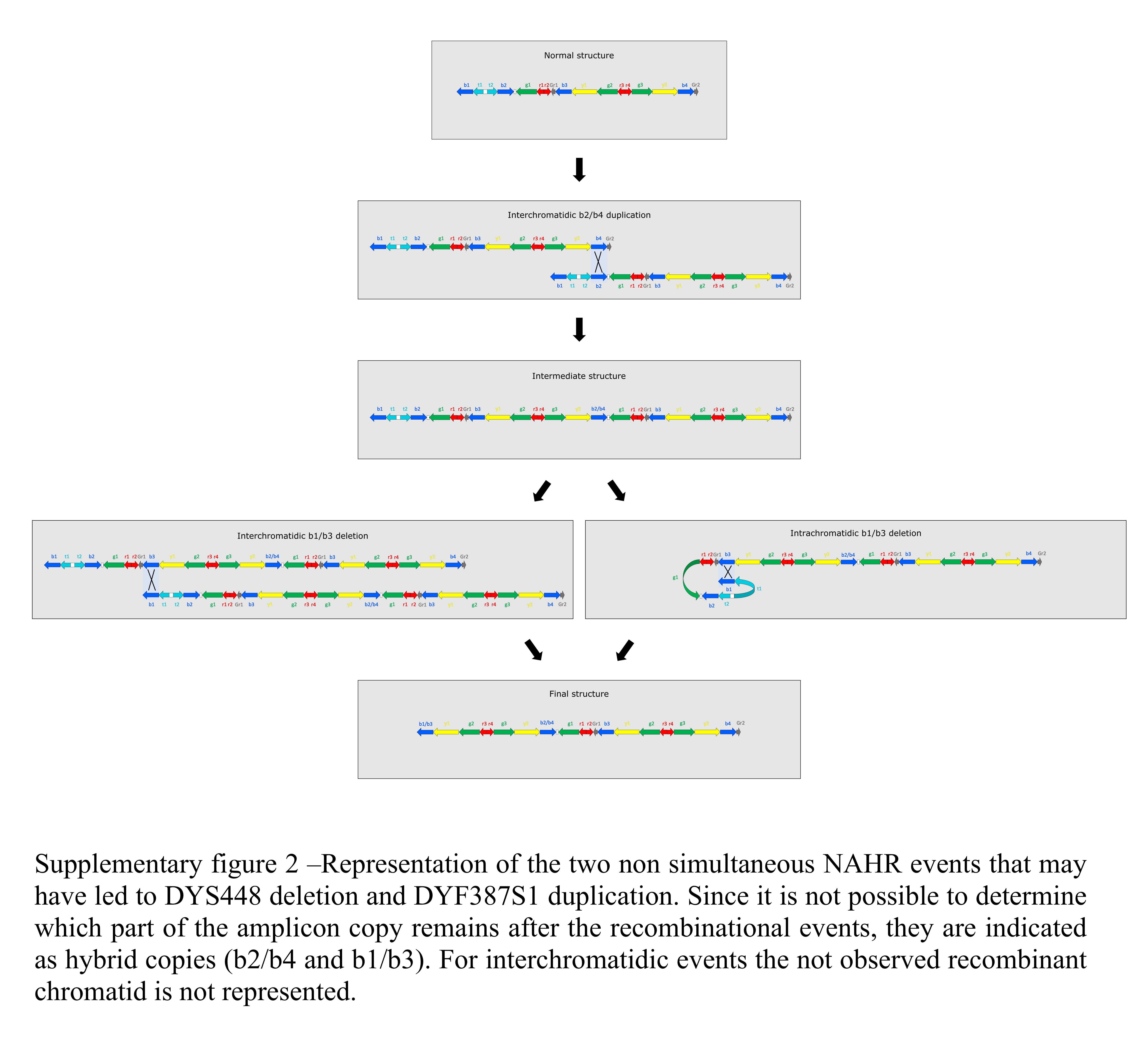

Supplement: Supplementary file 3 [file Image_2.JPEG]
